# Improvement of functional outcome for patients with newly diagnosed grade 2 or 3 gliomas with co-deletion of 1p/19q – IMPROVE CODEL: the NOA-18 trial

**DOI:** 10.1186/s12885-022-09720-z

**Published:** 2022-06-13

**Authors:** A. Wick, A. Sander, M. Koch, M. Bendszus, S. Combs, T. Haut, A. Dormann, S. Walter, M. Pertz, J. Merkle-Lock, N. Selkrig, R. Limprecht, L. Baumann, M. Kieser, F. Sahm, U. Schlegel, F. Winkler, M. Platten, W. Wick, T. Kessler

**Affiliations:** 1 Neurology Clinic and National Centre for Tumour Diseases, University Hospital Heidelberg, Heidelberg, Germany; 2 Institute of Medical Biometry and Informatics, University of Heidelberg, Heidelberg, Germany; 3 Department of Neuroradiology, University Hospital Heidelberg, Heidelberg, Germany; 4 Department of Radiation Oncology at the Klinikum Rechts der Isar, Technical University Munich, Munich, Germany; 5 Department of Neurology, University Hospital Knappschaftskrankenhaus Bochum, Ruhr University Bochum, Bochum, Germany; 6 Coordination Centre for Clinical Trials (KKS), Medical Faculty & University Hospital Heidelberg, Heidelberg, Germany; 7 Department of Neuropathology, University Hospital Heidelberg, DKTK and CCU Neuropathology, DKFZ, Heidelberg, Germany; 8 German Cancer Consortium (DKTK), Clinical Cooperation Unit Neurooncology, German Cancer Research Center (DKFZ), Heidelberg, Germany; 9 DKTK, Clinical Cooperation Unit Neuroimmunology and Brain Tumor Immunology, DKFZ, Heidelberg, Germany; 10 Department of Neurology, Medical faculty, MCTN, University of Heidelberg, Mannheim, Germany; 11 Neurology Clinic, University of Heidelberg & CCU Neurooncology, DKFZ, Im Neuenheimer Feld 400, D-69120 Heidelberg, Germany

**Keywords:** Qualified overall survival (qOS), Neurocognition, Procarbazine, CCNU, vincristine (PCV), CCNU and Temozolomide for Glioma (CETEG), Health-related quality of life (HRQoL), Oligodendroglioma

## Abstract

**Background:**

Given the young age of patients with CNS WHO grade 2 and 3 oligodendrogliomas and the relevant risk of neurocognitive, functional, and quality-of-life impairment with the current aggressive standard of care treatment, chemoradiation with PCV, of the tumour located in the brain optimizing care is the major challenge.

**Methods:**

NOA-18 aims at improving qualified overall survival (qOS) for adult patients with CNS WHO grade 2 and 3 oligodendrogliomas by randomizing between standard chemoradiation with up to six six-weekly cycles with PCV and six six-weekly cycles with lomustine and temozolomide (CETEG) (*n* = 182 patients per group accrued over 4 years) thereby delaying radiotherapy and adding the chemoradiotherapy concept at progression after initial radiation-free chemotherapy, allowing for effective salvage treatment and delaying potentially deleterious side effects. QOS represents a new concept and is defined as OS without functional and/or cognitive and/or quality of life deterioration regardless of whether tumour progression or toxicity is the main cause. The primary objective is to show superiority of an initial CETEG treatment followed by partial brain radiotherapy (RT) plus PCV (RT-PCV) at progression over partial brain radiotherapy (RT) followed by procarbazine, lomustine, and vincristine (PCV) chemotherapy (RT-PCV) and best investigators choice (BIC) at progression for sustained qOS. An event concerning a sustained qOS is then defined as a functional and/or cognitive and/or quality of life deterioration after completion of primary therapy on two consecutive study visits with an interval of 3 months, tolerating a deviation of at most 1 month. Assessments are done with a 3-monthly MRI, assessment of the NANO scale, HRQoL, and KPS, and annual cognitive testing. Secondary objectives are evaluation and comparison of the two groups regarding secondary endpoints (short-term qOS, PFS, OS, complete and partial response rate). The trial is planned to be conducted at a minimum of 18 NOA study sites in Germany.

**Discussion:**

qOS represents a new concept. The present NOA trial aims at showing the superiority of CETEG plus RT-PCV over RT-PCV plus BIC as determined at the level of OS without sustained functional deterioration for all patients with oligodendroglioma diagnosed according to the most recent WHO classification.

**Trial registration:**

Clinicaltrials.govNCT05331521. EudraCT 2018–005027-16.

## Background

Oligodendrogliomas in the novel edition of the Central Nervous System (CNS) World Health Organization (WHO) classification are now molecularly defined by isocitrate dehydrogenase (IDH)1 or IDH2 mutations and 1p/19q codeletion [1]. The prognosis of these molecularly defined tumors is to be determined in new series since survival data from older histology-based studies and population-based registries are confounded by the inclusion of 20–70% not molecularly matching patient subsets. Also, investigations on the optimal treatment mainly focus on targeting the IDH mutation, but also on optimizing the cytotoxic therapy. An extensive, but safe surgery is associated with improved outcomes as is the addition of chemotherapy with procarbazine, CCNU (lomustine), and vincristine (PCV) to partial brain radiotherapy [2]. However, the exact timing of postsurgical therapy especially for tumours of the WHO grade 2 and acknowledging some variability in grading as well as the choice of chemotherapy, temozolomide instead of PCV (CODEL: NCT00887146 randomizing CNS WHO grade 2 and 3 oligodendrogliomas to chemoradiotherapy with PCV or with temozolomide) or the need for primary radiotherapy are subjects of clinical studies (POLCA: NCT02444000 randomizing patients with newly diagnosed CNS WHO grade 3 oligodendrogliomas to standard chemoradiotherapy with PCV or PCV alone).

The discovery of a disease-defining [1] and prognostically favourable point mutation in *IDH1* codon 132 [3] has considerably altered our understanding of glioma biology [4]. The mutation results in a neomorphic enzymatic capacity to produce 2-hydroxyglutrate from α-ketoglutarate. Small molecule inhibitors with distinct specificities for activating IDH1-R132X/− 2 mutations have been designed and tested in early clinical trials. So far data are limited to early, uncontrolled studies demonstrating that (i) BAY1436032 has been well-tolerated and showed evidence of target inhibition and durable objective responses in a small subset of subjects with progressive grade 2 gliomas [5], (ii) ivosidenib showed an overall response rate in the MRI of 3% and stable disease in 86% in advanced non-enhancing gliomas while those with enhancing gliomas showed no objective responses and stability in 45% [6] as well as (iii) a phase I study of vorasidenib in non-enhancing progressive grade 2 gliomas showed an objective response rate of 18% (one partial remission and two minor responses) and stable disease in 73% [7]. These studies prompted further evaluations, including a phase III trial (INDIGO, NCT04164901). The primary aim of INDIGO is to prolong the time from diagnosis after surgery to the need for subsequent cytotoxic treatments in patients with residual or recurrent grade 2 gliomas with an IDH1 or IDH2 mutation between 1 and 5 years after the last surgery.

The present NOA-18 / IMPROVE CODEL protocol has been developed to reduce the burden of cytotoxic therapies and focuses on patient-centered outcomes. It is driven by the conviction that because of their relatively good prognosis and their vulnerability to therapy, treatment concepts for oligodendrogliomas need to be assessed for efficacy and impact on quality of life, cognition, and neurological function. Therefore, NOA-18 integrates patient-centered outcomes and considers late adverse effects. NOA-18 aims at improving qualified overall survival (qOS) for adult patients with CNS WHO grade 2 and 3 oligodendrogliomas by randomizing between standard chemoradiation with PCV and lomustine and temozolomide (CETEG). The experimental arm delays radiotherapy and adds chemoradiotherapy only at progression after the initial radiation-free chemotherapy, allowing for an effective salvage treatment but delaying potentially deleterious side effects. qOS represents a new concept and is defined as OS without functional and/or cognitive and/or quality of life deterioration regardless of tumor progression or toxicity being the main cause. In addition to the patient-centered outcome parameters, the trial is regarded as attractive for NOA expert sites and patients, because of the well-known treatments and hence limited additional burden. There is no larger competing trial open at present.

## Methods/design

### Trial design and overview

Despite the relatively good prognosis for patients with CNS WHO grade 2 and grade 3 oligodendrogliomas, the disease is non-curable and the burden of the disease and treatment is considerable. Standard postsurgical treatment differs only in the dosing of radiotherapy. Therefore, offering the same trial for patients with grade 2 and grade 3 oligodendrogliomas is straightforward and establishes the most homogenous trial population, potentially allowing for meaningful further subgrouping, i.e. to decipher patients with a very favourable course of the disease and also unexpected early progressors. However, the most critical problem for oligodendrogliomas concerns the considerable undesirable effects of therapeutic interventions on long-term Health-related Quality of Life (HRQoL), and cognitive and functional outcomes [8–10]. Although these considerations are regarded as critical by caregivers, patients (and their advocates) as well as relatives, all parties also agree that the mere life span and also any option for long-term survival, including curing the disease is also substantial, and needs to be weighed against the risks. Therefore, it is critical and reassuring for the proposed trial design that there is no difference in the median overall survival (OS) of patients with oligodendrogliomas initially treated with chemotherapy alone (10.5 years) or RT plus chemotherapy (8.4 years) [11]. The long-term analysis of the NOA-04 trial shows that temozolomide is similar to PCV and radiotherapy [12]. Median OS in patients with oligodendrogliomas is > 10 years with alkylating chemotherapy, implying no relevant hazard to the life span produced by initial alkylating monotherapy.

Patient-centered endpoints gain relevance in a disease with a reasonable prognosis but still incurable outcome. With this in mind, a transient functional decline at progression, which is manageable with a second surgery, steroids, and salvage therapy (with radiotherapy-PCV in the current concept), is not considered an event in the current trial, if the duration is ≤3 months. A replacement of procarbazine with temozolomide seems pharmacologically and clinically meaningful and the combination of lomustine plus temozolomide is a good combination scheme in the brain tumour population [13].

### Trial diagram

**Fig. 1:**
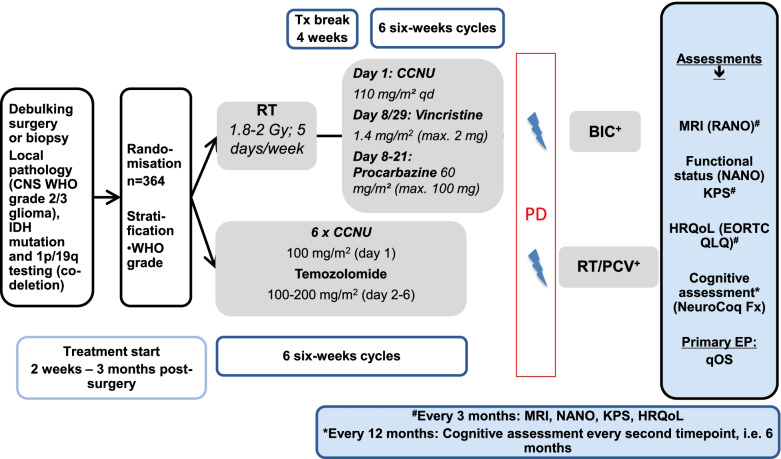
Trial summary of IMPROVE CODEL/NOA-18. *RT:* For CNS WHO grade 3 oligodendroglioma: Radiotherapy is performed as 33 fractions of 1.8 Gy for a total dose of 59.4 Gy. One fraction is given daily five days per week for about 6 to 7 weeks. For CNS WHO grade 2 oligodendroglioma, radiotherapy is performed as 28 fractions of 1.8 Gy for a total dose of 50.4/54 Gy for over approximately 5–6 weeks. *Tx Break:* Rest period is 4 weeks long (± 2 weeks) total. *PCV:* PCV chemotherapy, cycles are about 6 weeks long each. Day 1: CCNU 110 mg/m^2^ orally (capped at 200 mg); Days 8 and 29: vincristine 1.4 mg/m^2^ i.v. (capped at 2 mg); Days 8 to 21: procarbazine 60 mg/m^2^ orally (capped at 100 mg). *CETEG: CCNU/temozolomide chemotherapy cycles are 6 weeks long. Day 1: CCNU 100 mg/m*^*2*^ *orally* (capped at 200 mg)*; Days 2–6: Temozolomide 100 mg/m*^*2*^ *(cycle 1) with dose escalation in 50 mg/m*^*2*^ *steps according to toxicity in subsequent cycles.* ^*+*^*Therapy at progression is as suggested at the time of the protocol preparation. It is reasonable that patients without prior radiotherapy will undergo radiotherapy; it is also very likely that PCV will be used as adjunct chemotherapy if bone marrow reserve allows and if there are no safety concerns from the initial CETEG treatment. The treatment at progression in the standard arm is less predictable; there may be an option for second radiotherapy or even reuse of the full radiochemotherapy regimen as it had been given at diagnosis. The trial will therefore continue to assess any endpoint-relevant scans and scales and also list the secondary treatments, but not attempt a full registration of the treatment, which is considered standard and not driven by trial requirements*

The flow of the trial is depicted in Fig. 1. A list of study sites can be obtained from the coordinating investigator and will be published at clinicaltrials.gov.

### Objectives and hypothesis

#### Impact of the trial

NOA-18 aims at improving qOS by delaying radiotherapy and adding a powerful radiotherapy/chemotherapy concept at progression after initial radiation-free chemotherapy treatment, allowing for effective salvage treatment and delaying potentially deleterious side effects. qOS represents a new concept and is defined as OS without functional and/or cognitive and/or quality of life deterioration regardless of whether tumour progression or toxicity is the main cause. Therefore, an objective progression in the MRI alone does not represent an event for the endpoint. The present NOA trial aims at showing the superiority of chemotherapy plus radiotherapy-PCV over radiotherapy-PCV plus best investigators choice (BIC) as determined at the level of overall survival without sustained functional deterioration. Further, this trial will provide a major opportunity to elucidate the molecular basis of a better prognosis for this subgroup of patients with a yet incurable disease.

#### Primary objective

To show superiority of initial temozolomide plus lomustine (CETEG) chemotherapy followed by partial brain radiotherapy (RT) plus PCV (RT-PCV) at progression over partial brain radiotherapy (RT) followed by procarbazine, lomustine, and vincristine (PCV) chemotherapy (RT-PCV) and BIC at progression for qualified overall survival (qOS).

#### Secondary objectives

Evaluation and comparison of the two groups regarding secondary endpoints (short-term qOS, PFS, OS, complete and partial response rate).

#### Exploratory objectives

The trial will allow the assessment of new biomarkers.

### Patient selection

#### Main inclusion criteria

- Histologically confirmed, newly diagnosed CNS WHO Grade 2 or 3 oligodendroglioma.
- Tumour carries an *isocitrate dehydrogenase* (*IDH*) mutation (determined by immunohistochemistry (IHC) and/or deoxyribonucleic acid (DNA) sequencing).
- Tumour is co-deleted for 1p/19q (determined by genome-wide hybridization array, fluorescence in situ hybridization (FISH), multiplex ligation-dependent probe amplification (MLPA), or other appropriate methods).
- Open biopsy or resection.
- Age: ≥18 years.
- Karnofsky Performance Status (KPS) ≥60%.
- Life expectancy > 6 months.
- Availability of formalin-fixed paraffin-embedded (FFPE) or fresh-frozen tissue and ethylenediaminetetraacetic acid (EDTA) blood for biomarker research.
- Standard magnetic resonance imaging (MRI) ≤ 72 post-surgery according to the present national and international guidelines.
- Craniotomy or intracranial biopsy site must be adequately healed.
- ≥ 2 weeks and ≤ 3 months from surgery without any interim radio- or chemotherapy or experimental intervention.
- Willing and able to comply with regular neurocognitive and health-related quality of life tests/*questionnaires.*
- Indication for postsurgical cytostatic/−toxic therapy.
- Written Informed consent after a detailed oral explanation by a trial physician.
- Female patients with reproductive potential have a negative pregnancy test (serum or urine) within 6 days before the start of therapy. Female patients are surgically sterile or agree to use adequate contraception during the period of therapy and 6 months after the end of study treatment, or women have been postmenopausal for at least 2 years^1^.
- Male patients are willing to use contraception^2^.

#### Main exclusion criteria

- Participation in other ongoing interventional clinical trials.
- Insufficient tumour material for molecular diagnostics.
- Inability to undergo MRI.
- Lack of legal capacity
- Abnormal (≥ Grade 2 CTCAE v5.0 laboratory values for hematology (Hb, WBC, neutrophils, or platelets), liver (serum bilirubin, ALT, or AST), or renal function (serum creatinine).
- Active tuberculosis, HIV infection or active Hepatitis B (HBV) or Hepatitis C (HCV infection or active infections requiring oral or intravenous antibiotics or that can cause severe disease and pose a severe danger to lab personnel working on patients’ blood or tissue (e.g., rabies).
- Any prior anti-cancer therapy or co-administration of anti-cancer therapies other than those administered/allowed in this study. History of low-grade glioma that did not require prior treatment with chemotherapy or radiotherapy is not an exclusion criterion.
- Immunosuppression, is not related to prior treatment for malignancy.
- History of other malignancies (except for adequately treated basal or squamous cell carcinoma or carcinoma in situ) within the last 5 years unless the patient has been disease-free for 5 years.
- Any clinically relevant concomitant disease (including hereditary fructose intolerance) or condition that could interfere with, or for which the treatment might interfere with, the conduct of the study or the absorption of oral medications or that would, in the opinion of the Coordinating Investigator, pose an unacceptable risk to the patient in this study.
- Any psychological, familial, sociological, or geographical condition potentially hampering compliance with the study protocol requirements and/or follow-up procedures; those conditions should be discussed with the patient before trial entry.
- Pregnancy or breastfeeding.
- History of hypersensitivity to the investigational medicinal product or any drug with a similar chemical structure or any excipient present in the pharmaceutical form of the investigational medicinal product.
- QTc time prolongation > 500 ms.
- Patients under restricted medication for procarbazine, lomustine, vincristine, and temozolomide
- Liver disease characterized by:
  - Alanine aminotransferase or aspartate aminotransferase (≥ Grade 2 CTCAE v5.0) confirmed on two consecutive measurements
  OR
  - Impaired excretory function (e.g., hyperbilirubinemia) or synthetic function or other conditions of decompensated liver disease such as coagulopathy, hepatic encephalopathy, hypoalbuminemia, ascites, and bleeding from oesophageal varices (≥ Grade 2 CTCAE v5.0)
  OR
  - Acute viral or active autoimmune, alcoholic, or other types of acute hepatitis
- Known uncorrected coagulopathy, platelet disorder, or history of non-drug-induced thrombocytopenia.
- History of autoimmune disease, including but not limited to myasthenia gravis, myositis, autoimmune hepatitis, systemic lupus erythematosus, rheumatoid arthritis, inflammatory bowel disease, vascular thrombosis associated with antiphospholipid syndrome, Wegener’s granulomatosis, Sjögren’s syndrome, Guillain-Barré syndrome, multiple sclerosis, vasculitis, or glomerulonephritis; autoimmune-related hypothyroidism (patients on a stable dose of thyroid replacement hormone are eligible for this study) and type I diabetes mellitus (patients on a stable dose of insulin regimen are eligible for this study).
- Vaccination with life vaccines during treatment and 4 weeks before the start of treatment.
- Existing neuromuscular diseases, especially neural muscular atrophy with segmental demyelination (a demyelinating form of Charcot-Marie-Tooth syndrome)
- Chronic constipation and subileus
- Combination treatment with mitomycin (risk of pronounced bronchospasm and acute shortness of breath)
- Hypersensitivity to dacarbazin

### Interventions

The control intervention consists of radiotherapy at 50.4/54 Gy (CNS WHO grade 2 oligodendrogliomas)/59.4 Gy (CNS WHO grade 3 oligodendrogliomas) followed by six cycles of PCV according to the commonly used regimen and as a clinically indicated intervention best investigators choice (BIC) at progression.

**Fig. 2:**
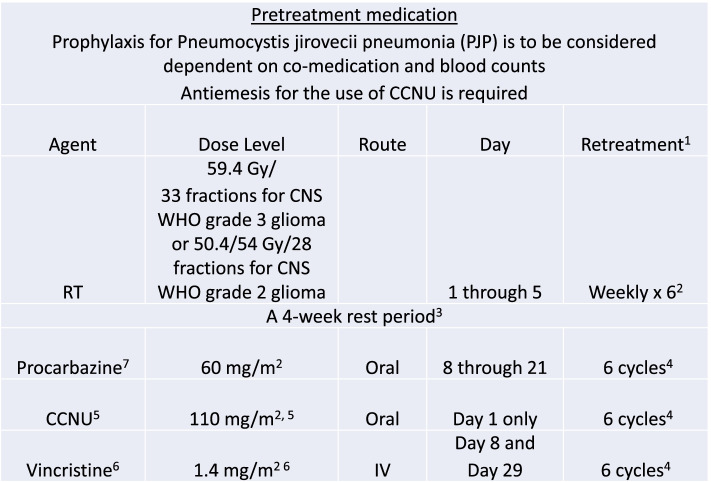
RT-PCV administration and dosing. ^1^Follow these procedures for pseudoprogression: a) if pseudoprogression is felt to be present, treatment should continue and functional imaging (i.e., MRI perfusion, spectroscopy) or pathological confirmation should be considered; b) if pseudoprogression is known to be present, continue treatment per study protocol; c) if tumor progression is present, the patient should be discontinued from the study; d) if equivocal, contact the study PI. ^2^For CNS WHO grade 3 oligodendroglioma phase 1 RT is about 6 to 7 weeks long total. For CNS WHO grade 2 oligodendroglioma phase 1 RT is about 6 weeks. ^3^Phase 2 Rest Period is 4 weeks long (± 2 weeks) total. ^4^Phase 3 (chemotherapy cycles) are about 6 to 7 weeks long each. ^5^The maximum dose of CCNU (dose cap) is 200 mg. CCNU is administered at a dose of 110 mg/m^2^ body surface area calculated according to Du Bois once every six weeks. It is recommended to be taken at least 3 h after the last meal in the morning or the evening. An antiemesis using a 5HT3 antagonist or a comparable medication should be used one hour before CCNU. The capsules should be swallowed whole and not opened or dissolved. If the dose is missed it can be taken within 48 hours of the usual starting day, but the interval to the first dose of procarbazine needs to be maintained. ^6^The maximum dose (dose cap) of vincristine is 2 mg. ^7^Procarbazine is usually taken fasting in the morning or 3 hours after the last meal at any time at 2 capsules. There is no specific concomitant medication. Some patients may benefit from an antiemesis at the discretion of the investigator

This treatment arm is coined RT-PCV plus BIC (Fig. 2).

The experimental intervention consists of six 42-day cycles of lomustine plus temozolomide according to the presently used regimen (CETEG) [13] and as a clinically indicated intervention radiotherapy at 50.4/54 or 59.4 Gy often followed by six cycles of PCV according to the commonly used regimen or other chemotherapy at progression.

**Fig. 3:**
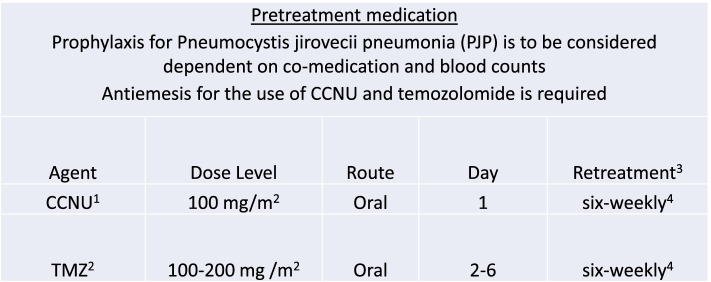
CETEG administration and dosing. ^1^ CCNU is administered at a dose of 100 mg/m^2^ body surface area calculated according to Du Bois once every six weeks. It is recommended to be taken at least 3 h after the last meal in the morning or the evening. An antiemesis using a 5HT3 antagonist or a comparable medication should be used one hour before CCNU. The capsules should be swallowed whole and not opened or dissolved. If the dose is missed it can be taken within 48 hours of the usual starting day, but the interval to the first dose of TMZ needs to be maintained. ^2^ The first cycle of temozolomide is administered at the dose of 100 mg/m^2^. It will be taken once daily (QD) at 100 mg per m^2^ body surface area calculated according to Du Bois on days 2–6 of a 42 days cycle, fasting in the morning. An antiemesis using a 5HT3 antagonist or a comparable medication should be used one hour before temozolomide. The capsule should be swallowed whole and not opened or dissolved. If a dose is missed it can be taken within 6 hours of the usual morning dose. If the time is greater than 6 hours than the regular time or the patient vomits the dose, the patient should wait and take the next dose. The dose is escalated to 150 mg/m^2^ and 200 mg/m^2^ as of subsequent cycles in the absence of toxicity. ^3^ Follow these procedures for pseudoprogression: a) if pseudoprogression is felt to be present, treatment should continue and functional imaging (i.e., MRI perfusion, spectroscopy) or pathological confirmation should be considered; b) if pseudoprogression is known to be present, continue treatment per study protocol; c) if tumor progression is present, the patient should be discontinued from the study; d) if equivocal, contact the study PI. ^4^Treatment cycles are about 6 weeks long each

This arm is coined CETEG plus RT-PCV (Fig. 3).

Temozolomide and lomustine (CCNU) are approved for gliomas. PCV is used according to current guidelines (AWMF 030/099 and EANO Guidelines [2]). Details for dose adjustments are provided in the Supplementary Information.

The duration of intervention per patient is approximately 12 months dependent on potential delays due to toxicity or patients’ wish. The follow-up per patient is at a minimum of 5 years and a maximum of 9 years. There is insurance for trial participants covering harm inflicted by trial treatment.

### Adverse events

#### Definition

Adverse events will be collected throughout the study and assessed according to the NCI-CTCAE, version 5.0. AEs will be tabulated and compared between study arms.

#### Analysis

The analysis of the safety endpoints is based on the safety set, which comprises all patients who have received study medication at least once and patients will be considered as treated. The analysis includes calculation and comparison of the rates of adverse and serious adverse events and a graphical display of the time course.

#### Assessments

The assessment of safety will be mainly based on the frequency of adverse events and on the number of laboratory values that fall outside of pre-determined ranges and/or show prominent worsening from baseline. Frequencies of patients experiencing at least one AE will be displayed. The detailed information collected for each AE will include: A description of the event, whether the AE was serious, the intensity, the relationship to the study drug, actions taken, and the clinical outcome. Summaries of incidence rates (frequencies and percentages) of AEs by MedDRA System Organ Class (SOC) and Preferred Term will be prepared. Such summaries will be displayed for all AEs, AEs by intensity, and AEs by the relationship to the study drug. Summary tables will present the number of patients observed with AEs and the corresponding percentages. Summary tables will be prepared to examine the distribution of laboratory measures over time. Laboratory data will be summarized by presenting shift tables using normal ranges (baseline to most extreme post-baseline value) and by presenting summary statistics of raw data and changes from baseline values (means, medians, standard deviations, ranges). All proportions will be given along with Pearson-Clopper 95% confidence bounds.

### Data collection and handling

All findings including clinical and laboratory data will be documented by the investigator or an authorized member of the study team in the patient’s medical record and the case report form (CRF). The investigator at the clinical site is responsible for ensuring that all sections of the CRF are completed correctly and that entries can be verified against source data. The CRF has to be filled out according to the specified CRF Completion Guidelines. The correctness of entries in the CRF will be confirmed by the dated signature of the responsible investigator.

- For the following parameters, the CRF will serve as the source document: Karnofsky Performance Status.

Results of central disease assessment performed by the Central Neuropathology will be reported directly (e.g., electronically or by separate reporting sheets) to the Coordinating Center for Clinical Studies (KKS) Heidelberg.

All data will be reported pseudonymized. Patients will be actively motivated to adhere to study-related visits and documentation.

### Efficacy endpoints

#### Primary efficacy endpoint

The primary efficacy endpoint is overall survival without functional and/or cognitive and/or quality of life deterioration after completion of primary therapy over 90 days, coined qualified overall survival (qOS). Short-term qOS is defined as the time from randomization to

1. a confirmed cognitive deterioration of an individual test performance that is defined as follows: 1. A detriment of ≥1.5 standard deviations below the normative mean in two or more NeuroCog FX® subtests AND 2. Related to baseline: 90%-confidence intervals of NeuroCog FX® subtests indicate a clinically and statistically meaningful individual change (i.e., deterioration) in two or more NeuroCog FX® subtest raw scores [14]
2. or related to baseline: a decrease in the KPI from 100 or 90 to 70 or less, a decrease in KPI of at least 20 from 80 or less, or a decrease in KPS from any baseline to 50 or less. Fulfillment of one of these criteria is considered neurological deterioration unless attributable to comorbid events or changes in corticosteroid dose (van den Bent et al. 2011),
3. or related to baseline: a worsening of at least 10 points, which is the minimal clinically relevant difference, in at least one of the five selected domains of the HrQoL (global health status (GHS), physical functioning (PF), social functioning (SF), determined in the QLQ-C30 with higher scores indicate better HRQoL; communication deficits (CD) & motor dysfunction (MD) determined by QLQ-BN20 with lower scores indicate better HRQoL) [15],
4. or a decline in the NANO scale defined as a ≥ 2 level worsening from baseline within ≥1 domain or worsening to the highest score within ≥1 domain that is felt to be related to underlying tumor progression and not attributable to a comorbid event or change in concurrent medication [16],
5. or death due to any cause,

whatever occurs first. An event concerning a sustained qOS (primary endpoint) is then defined as a functional and/or cognitive and/or quality of life deterioration (as described above) on 2 consecutive study visits with an interval of 3 months (90 days), tolerating a deviation of at most 28 days. Thus, after the first decline in any of the above-mentioned assessments the next study visit has to be within 62 to 118 days. The deterioration can be observed in the same score at the two visits but can be on different items as well to have qOS reached. If functional and/or cognitive deterioration is observed at one visit and the patient drops out or is lost to follow up after this visit, this will be handled as an event. Patients still alive without one of the above-defined functional and/or cognitive and/or quality of life deterioration criteria at a study visit or lost to follow-up at the time of study end will be censored at the last date they were known to be alive without deterioration.

If one or more of the assessments that are due every 3 months (MRI, NANO, KPS, HRQoL) are missing and a new test is only done at the regular next visit,

- a stable or improved result will account as stable and no action is necessary,
- a worse result in that missing domain compared to the last data point available (> 3 months) will result in a progression and backdating for the primary endpoint to the date of the missed examination
- a worse result in another than the missing domain compared to the last data point available (≤ 3 months) will result in a procedure as outlined in 2.

**Fig. 4:**
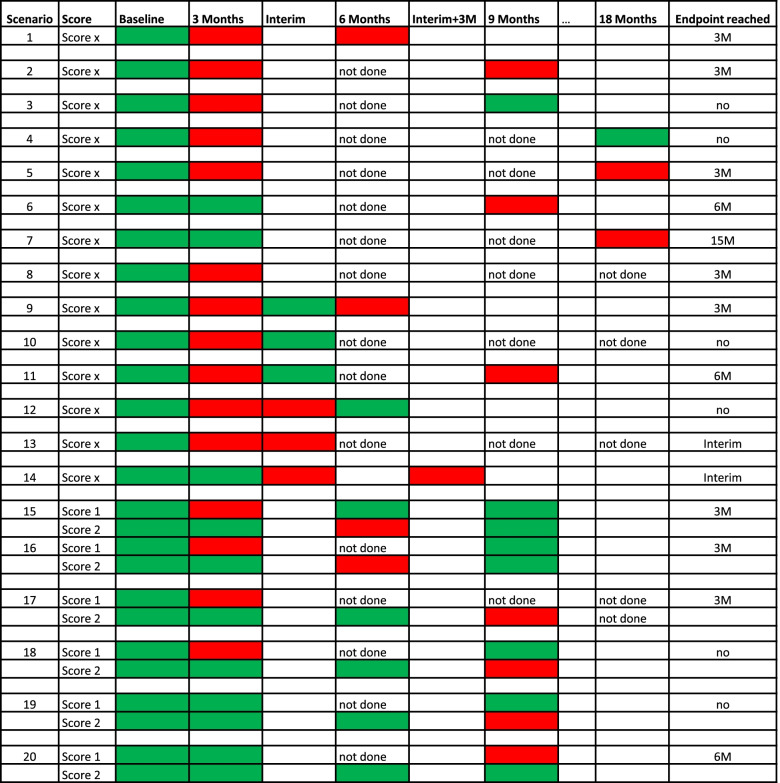
Definition of the primary endpoint (including handling of missing values/visits). Green means that there is no decline, and red means that there is a decline that is relevant in terms of the primary endpoint

An outline of the considerations for the management of potentially missing values is provided in Fig. 4.

#### Secondary efficacy endpoint

The secondary efficacy endpoints are evaluation and comparison of the two groups regarding short-term qOS, PFS, OS, and complete and partial response rate.

- short-term qOS is defined as qOS as described above but neglects the subsequent time interval of 3 months (90 days). Patients still alive without one of the above-defined functional and/or cognitive deterioration criteria at a study visit or lost to follow-up will be censored at the last date they were known to be alive without deterioration.
- OS is defined as the time from randomization until death due to any cause. Patients still alive will be censored at the last date they were known to be alive.
- PFS is defined as the time from randomization to the day of first documentation of clinical or radiographic tumor progression or death of any cause (whichever occurs first). Patients without a PFS event will be censored at the last disease assessment showing no progression or at baseline if the patient has no post-baseline disease assessments. PFS analysis on MRI according to a standardized MR protocol will be based on the central disease assessment by the central reading in the Dept. of Neuroradiology Heidelberg [17].

1. Response rate is defined as complete and partial responses according to Response Assessment in Neuro-Oncology (RANO) criteria, which integrate MRI changes (based on FLAIR images and contrast enhancement on T1-w images), clinical findings, and changes in steroid use (RANO, [17]).

Exploratory imaging endpoints will include multiparametric AI-based analysis of MR data a) to assist PFS assessment by visual inspection according to RANO criteria, b) early differentiation between radiation effects and tumor progression, and c) defining imaging patterns that may predict response to therapy.

### Statistical considerations

#### Sample size calculation

The sample size calculation is based on the primary efficacy endpoint qOS. The OS in both groups (for patients with CNS WHO grade 2 or 3 oligodendrogliomas) is to be expected non-different at the end of the trial [11, 12, 18–21]. The 5-year rate of qOS is expected to be 85% in the experimental intervention group [20]. Because of a higher rate of cognitive and/or functional and/or quality of life decline to be expected in the control group, the 5-year rate of qOS is assumed to be 75% in the control group [10]. An absolute improvement of 10% in the 5-year rate of qOS is clinically relevant and achievable. For exponentially distributed survival times, this treatment group difference corresponds to a hazard ratio of 0.565 (experimental vs. control). A group-sequential design is applied with one interim analysis after half of the expected number of events using the stopping rule according to O’Brien and Fleming [22]. To detect a hazard ratio of 0.565 at a significance level of 5% (two-sided) with a power of 80%, a total of 97 events is required for the entire trial. With an accrual period of 4 years and a follow-up period of at least 5 years, 364 patients must be included (182 per group). The interim analysis will then take place approximately 5¼ years after the accrual start. These calculations are based on the approximation formula by Schoenfeld [23, 24] for the log-rank test and the two-stage O’Brien and Fleming design (ADDPLAN 6.0).

#### General methodology

The statistical analysis will be carried out by the responsible trial statistician at the Institute of Medical Biometry at the University of Heidelberg using the current version of the SAS statistical software (SAS Institute Inc., Cary, NC, USA). The interim analysis will be done as soon as half of the expected number of events concerning the primary efficacy endpoint qOS were documented. The final analysis will be done as soon as the database has been declared to be complete and accurate and has been locked. Detailed descriptions of the planned analyses and reporting will be defined in the statistical analysis plan, which must be written and authorized by the trial statistician and the Coordinating Investigator before the interim analysis started.

Demographic and other Baseline Characteristics will be summarized per intervention group. Statistical methods are used to assess the homogeneity of intervention groups. There will be counting of the absolute and relative frequencies (percentages) for categorical variables. The Chi-square test will be performed to compare frequencies between groups.

Continuous variables and changes (differences) from the baseline assessment will be summarized using standard measures of central tendency and dispersion. This will include the number of observations, number of missing values, mean, standard deviation, minimum, maximum, median, and interquartile range. According to the distribution of the variable parametric or non-parametric tests will be applied to compare the groups.

#### Analysis of the primary endpoint

The confirmatory analysis of the primary efficacy endpoint will be conducted in the modified intention-to-treat (mITT) set, which comprises all randomized patients who received at least one dose of study treatment and allocates the patients in the treatment group they were assigned to by randomization. The confirmatory test for treatment group difference concerning qOS will be done using a Cox proportional hazards model with cofactors treatment group, CNS WHO grade (2/3), the extent of resection (biopsy, incomplete/complete), and *MGMT* status (promoter methylation yes/no) and the covariate age. Furthermore, center-specific random intercepts will be specified.

A group-sequential design with a stopping rule according to O’Brien and Fleming (O’Brien and Fleming, 1979) is applied with one interim analysis after half of the expected number of events has occurred. The interim analysis offers the option to stop the trial for superiority but is expected to take place approximately 1¼ years after the end of the 4 years of accrual. The overall two-sided type I error rate is limited by 5%. The corresponding two-sided 95% confidence interval for the hazard ratio of the experimental versus the control intervention is calculated considering the sequential nature of the design.

The primary efficacy endpoint qOS will be displayed for each intervention group based on the Kaplan-Meier estimates as well as on the estimates obtained from the Cox regression model. For the primary endpoint, there will be no missing data problem as missing information is handled within the definition of qOS. Missing data concerning the cofactors WHO grade, the extent of resection, and *O6-methylguanine-DNA-methyltransferase (MGMT)* promoter methylation status will be replaced by using multiple imputations.

Median survival with 95% confidence intervals and survival rates will be calculated. In addition to the mITT set, the primary efficacy endpoint is evaluated in the per-protocol set, which comprises all patients of the mITT set without major protocol deviations, as well as inappropriate subgroups as sensitivity analyses.

#### Analysis of secondary endpoints

Descriptive methods will be used for the analysis of the secondary efficacy endpoints. Time-to-event endpoints will be analyzed the same way the primary endpoint is analyzed. Binary secondary endpoints will be analyzed using logistic regression models, and ordinal secondary endpoints (e.g., RANO scale) using an ordinal logistic regression model. Appropriate summary measures of the empirical distributions as well as descriptive *p*-values will be calculated. Additionally, sensitivity analyses will be conducted for different populations (*per-protocol* set, appropriate subgroups). Exploratory analyses will be performed to identify and investigate potential prognostic factors for an intervention effect. Missing values in secondary endpoints will not be imputed. The details will be laid out in the Statistical Analysis Plan.

### Data Safety Monitoring Committee (DSMC)

In case of any irregularities, e.g., concerning the frequency or type of reported SAE the principal investigator will inform the members of the independent DSMC without delay. At least once every 12 months, the DSMC will receive a written safety report and have a meeting (or telephone conference) to discuss the report. The first meeting will take place after the first year. The DSMC will also receive the results of the interim analysis. The members of the DSMC then report the result of the benefit/risk assessment to the principal investigator and will give appropriate recommendations concerning the continuation of the trial. The working procedures of the DSMC will be recorded in the DSMC charter of the trial.

## Discussion

There are interesting and promising developments for patients with oligodendrogliomas. These include active chemoradiotherapy as standard-of-care, inhibition of IDH as a disease-specific intervention targeting the neomorphic function of the mutated IDH and potential metabolic consequences as well as the impact on the regulation of DNA-replication [5–7], and immunotherapeutic interventions also targeting a disease-specific lesion that is the most common IDH mutation (IDH1R132H) [25]. At present, IDH vaccines are in early, IDH inhibitors are in later development, but so far in a postsurgical pre-genotoxic phase of the disease treatment, aiming at preventing disease progression.

Despite the ongoing efforts, there are relevant challenges regarding the optimal treatment of patients with oligodendrogliomas. Should all patients with grade 2 and 3 oligodendrogliomas be treated with radiotherapy and chemotherapy? Is there any chance to achieve the same benefit with chemotherapy with PCV (or temozolomide or a combination of both) alone? PCV is the chemotherapy in the current standard chemoradiotherapy for patients with oligodendrogliomas. Can PCV be safely and effectively replaced by temozolomide? Considering the overall favourable prognosis of more than a decade median overall survival time, in which grounds do we make a decision for a treatment? And how can the efficacy and toxicity, specifically be weighed against each other? The ongoing POLCA (NCT02444000) trial focuses on the question of omitting radiation from the front-line treatment and using PCV alone.

NOA-18 proposes that CETEG might be the more attractive experimental regimen compared to PCV. It uses the more active temozolomide instead of procarbazine [26], leaves out vincristine, which may only minimally add to the efficacy of PCV, but certainly add (peripheral) neurotoxicity [27], and has shown activity in patients with newly diagnosed glioblastoma (13). Even more importantly, the present trial consequently introduces a qualification of overall survival by using health-related quality of life, neurological function, and cognition relevant (patient-centered) data as endpoints.

## Data Availability

Not applicable. Sharing of data generated from this study will adhere to the Bundesministerium für Bildung und Forschung (BMBF) guidelines regarding sharing of data collected using federal funds. There will be extensive public dissemination of trial results to the expert and general community using publications, meetings, social media, and other ethically accepted ways of publication. Authorship will be granted to individuals dependent on their involvement in the trial adhering to the International Committee of Medical Journal Editors (ICMJE) guidelines.

## Abbreviations

AE: Adverse Events
BMBF: Bundesministerium für Bildung und Forschung
CD: Communication Deficits
CETEG: CCNU and Temozolomide for Gliomas
CNS: Central Nervous System
CTCAE: Common Terminology Criteria for Adverse Events
DKTK: German Cancer Consortium
DSMC: Data Safety Monitoring Committee
FFPE: Formalin-Fixed Paraffin-Embedded
FLAIR: Fluid-attenuated inversion recovery
GHS: Global Health Status
HrQoL: Health-related Quality of Life
IDH: Isocitrate Dehydrogenase
ICMJE: International Committee of Medical Journal Editors
KKS: Coordinating Center for Clinical Studies
KPS: Karnofsky Performance Status
MD: Motor Dysfunction
MGMT: O6-Methylguanine-DNA-Methyltransferase
mITT: modified Intention-To-Treat
MRI: Magnetic Resonance Imaging
NCI: National Cancer Institute
NOA: Neurooncology Working Group of the German Cancer Society
ORR: Overall Response Rate
OS: Overall Survival Rate
PCV: Procarbazine, CCNU (lomustine), Vincristine
PF: Physical Functioning
PFS: Progression-Free Survival Rate
POLCA: PCV Only in 1p/19q Codeleted Anaplastic Gliomas
qOS: qualified Overall Survival
RANO: Response Assessment for Neuro-Oncology
RT: Radiotherapy
SF: Social Functioning
SOC: Standard of Care
TMZ: Temozolomide
WHO: World Health Organization

## Footnotes

1 Acceptable method of contraception comprises barrier contraception combined with a medically accepted contraceptive method for the female patient or female partner (e.g. intrauterine device with spermicide, hormonal contraceptive since at least 2 months). Female patients must agree not to donate lactation during treatment and until 6 months after the end of the study treatment.

2 Patients should be advised to seek consultation on sperm conservation before treatment starts. Condoms with spermicidal jellies or cream) upon study entry and during the course treatment and 6 months after the end of treatment, have undergone vasectomy, or are practicing total abstinence. Their female partners of childbearing potential should also be advised to use contraception during this period. Sperm donation is not permitted for the same time interval.
